# Development and Evaluation of Cellulose/Graphene-Oxide Based Composite for Removing Phenol from Aqueous Solutions

**DOI:** 10.3390/polym15030572

**Published:** 2023-01-22

**Authors:** Naveen Kumar, Bijender Kumar, Himanshu Gupta, Anuj Kumar

**Affiliations:** 1 Department of Chemistry, S.D. College, Muzaffarnagar, MaaShakumbhari University, Saharanpur 251001, Uttar Pradesh, India; 2 Creative Research Centre for Nanocellulose Future Composites, InhaUniversity, 100, Inharo, Michuhol-gu, Incheon 22212, Republic of Korea; 3 Department of Chemistry, School of Sciences, IFTM University, Moradabad 244102, Uttar Pradesh, India; 4 School of Chemical Engineering, Yeungnam University, Gyeongsan 38541, Republic of Korea

**Keywords:** cellulose, graphene oxide, composite, phenol, adsorption

## Abstract

In this study, a graphene oxide/cellulose composite (GO–cellulose) was prepared usingcellulose and graphene oxide (GO) through ultrasonication, followed by the freeze-dried method. The Brunauer–Emmett–Teller (BET) specific surface area of GO–cellulose (~6.042 m^2^/g) was higher compared to cellulose (1.023 m^2^/g).The UV-Visible spectraindicated that the prepared GO–cellulose composite removedphenol efficiently from aqueous solutions with high adsorption power. The effectiveness of the composite for phenol adsorption was examinedunder diverse conditions.The results reveal that the composite optimally improved the adsorption at pH 7 with a dose of 0.125 g/30 L in about 40 min. The adsorption process showed that in optimum conditions, 86 ± 2% of phenol was removed in 40 min with an adsorption capacity of 6.192 mg g^−1^. The adsorption behavior was well fitted to the pseudo-second-order kinetic model and the Langmuir isotherms at all temperatures.The present study suggests that synthesized GO–cellulose is useful inthe removal of phenol pollutants from aqueous solutions.

## 1. Introduction

Recently, water quality standards have becomehighly significant due tothe discharge of dangerous contaminants into water bodies. One of the essential pollutants from this point of view is phenol and its derivatives. These pollutants’ primary sources are effluents from pharmaceuticals, coal, petrochemicals, and many other industries [1,2]. These phenolic compounds may adversely affect aquatic life and human health, such as causing diarrhea, impaired vision, severe skin damage, cardiovascular diseases, and serious gastrointestinal damage. Phenols may enter the body bydermal contact, ingestion, or inhalation, and are toxic even at lower concentrations [3,4]. It was reported that the levels of toxicitygenerally range between 9–25 mg/L for humans, animals, and other living organisms. As per the US Environmental Protection Agency, phenol isconsidered as a priority pollutant with a 0.1 mg/L permissible limit in wastewater [5].

It is necessary to eliminate these phenolic compounds from water. Several methods, such as chemical oxidation, photocatalytic degradation, microbial degradation, solvent extraction, and adsorption, have been developed to remove phenols and their derivatives from aqueous media [6]. Among them, adsorption is most popular due to its numerous advantages, e.g., higher removal efficiency, low adsorbent regeneration cost, low secondary pollution, low energy consumption, and easy operation [7,8]. Therefore, many studies focus on the adsorptive removal of phenol from aqueous systems. Many absorbent materials have explored to remove phenol from water, including activated carbon, activated alumina, silica gel, bagasse ash, wood charcoal, and bamboo charcoal. Among these adsorbents, waste biomass adsorbentsare especially of interest due to their low cost, renewability, and biodegradability [9,10,11,12]. The ground nut is a globallysignificantcrop worldwide, and large quantities of waste ground nut shells are generated yearly. Ground nut shells as cellulosic waste materials have been usedto convert useless raw materials into useful valuable adsorbents [13,14,15]. Cellulose is the most abundant natural polymer that meets severalrequirements, such asbeinglowcost, non-toxic, renewable, biodegradable, modifiable, and an excellent adsorbent [16]. It has received significant attention as a biomass adsorbent substrate. However, cellulose is difficult to dissolve in water as well as common solvents due to strong intra- and intermolecular hydrogen bonding and a high degreeof polymerization [17], which have become limitations for its application as an adsorbent in aqueous media. Thus, in recent years the functional modification of cellulose opened up a new way to develop a new cellulose adsorption system.

Graphene oxide (GO) is ideally present in two-dimensional sheets with carbon atoms in the monolayer. GO has various oxygen-containing functional groups, such as carboxyl, epoxides, ketones, alcohols, and hydroxyl groups, which result in noteworthy swelling and intercalationcharacteristics. Furthermore, due to oxygen-containing functional groups, GO is intensely hydrophilic and instantly disperses in water and further organic solvents [18,19]. Its vast surface area and large functional groups endow it with effective adsorption uses. Recently, GO has attracted much attention as an important constituent of new composite materials. It is believed that the incorporation of GO into some matrices forms a composite adsorbent with better adsorption capacity [20,21,22,23]. Based on adsorption characteristics of cellulose and GO, these were combined to prepareadsorbents with higheradsorption capacities.Miao et al. [24] applied Hummers methods using microcrystalline cellulose to prepare a graphene oxide/cellulose composite. The prepared composite has application value in hydrophobic substances. Chen et al. [25] developed nanocomposite films using cellulose and graphene in urea. The prepared composite was applied for sensing applications. Tao et al. [26] employed graphene oxide and cellulose nanocrystals to remove levofloxacin hydrochloride from aqueous media. Abd-Elhamid et al. [27] modified carboxymethyl cellulose with GO for enhancement of adsorption for Ce (III) and Nd (III). Hao et al. [28] prepared a GO–cellulose composite in ionic liquid for the adsorption of Ce (III). Chen et al. [29] utilized NaOH and urea to prepare a GO–cellulose hydrogel to absorb heavy metals from aqueous media.Only a few studies are available thatprepared GO–cellulose composites using waste groundnut peels. Furthermore, no studies have been found in the literature reporting the adsorption of phenol using a ground-nut-peel-derived composite of GO–cellulose.

In this paper, the cellulose was first isolated from waste ground nut shells; after that, GO was successfully incorporated into cellulose to synthesize a GO–cellulose composite using ultrasonication followed by the freeze-dried method. Subsequently, theprepared GO, cellulose, and GO–cellulose composite were characterized by varioustechniques. The surface morphology and structure properties of the GO–cellulose composite were characterized using FTIR, XRD, FE-SEM, EDS, TGA, and Brunauer–Emmett–Teller (BET) analysis. The adsorption performance of phenol onto the GO–cellulose composite was investigated under different experimental settings, such as contact time, amount of adsorbent, pH, and temperature effect, and the optimized reaction conditions for phenol adsorption were also studied.

## 2. Materials and Methods

### 2.1. Materials

Ground nuts were taken from the local market of Muzaffarnagar (UP), India. Dry ground nut shells were utilized without any further treatment. All chemicals were used as received and included the following: graphite flakes (Merck), H_2_SO_4_ (Merck), KMnO_4_ (Merck), HNO_3_ (Merck), sodium hypochlorite (Himedia, India), aqueous 30 % (H_2_O_2_)(Rankem, India), NaOH (Rankem, India), HCl(Rankem, India), and phenol (LobaChemie, India)

### 2.2. Extraction of Cellulose from a Ground Nut Shell

The pure cellulose from ground nut shells was extracted according to the previously reported methods [30,31]. First, the groundnut shells were washed with distilled water several times and then dried.Further, dried ground nut shellsweregroundand sieved over 40–80 mesh screens to keep the size uniformity of powdered ground nut shells. Then, 20 g of ground nut shells powder was mixed in 500 mL of 2 % *w*/*v* sodium hydroxide and digested for 6 h at 80 °C in a water bath. After washing and filtration, the mixture was bleached with 500 mL of a 1.5% (*w*/*v*) sodium hypochlorite solution for 15 min at 80 °C. The material was washed sufficiently with deionized water, and the procedureof purification was repeated.The resulting α-cellulose was washed several times with deionized water and then dried at 70 °C overnight.

### 2.3. Preparation of Graphene Oxide (GO)

The GO wassynthesizedby flake graphite using the modified Hummers method [32]. In brief, 1.0 g of the graphite flakes wasadded to a mixture of nitric acid (10.0 mL) and sulfuric acid (15.0 mL) in a round-bottom flask under stirring at ambient temperature. Then, 3.0 g of KMnO_4_ was supplemented to the above reaction mixture under stirring in an ice bath to keep the suspension temperaturebelow 20 °C. Further, the reaction mixture was stirred at room temperature for 10 h. After that, H_2_O_2_ (30%, 15.0 mL) was added slowly to remove the permanganate excess. The mixture was filtered and washed with 250 mL of 2% HCl aqueous solution and deionized water to remove ions and acid. The resulting powder was dried and stored for further use.

### 2.4. Preparation of GO–Cellulose Composite

**Scheme 1:**
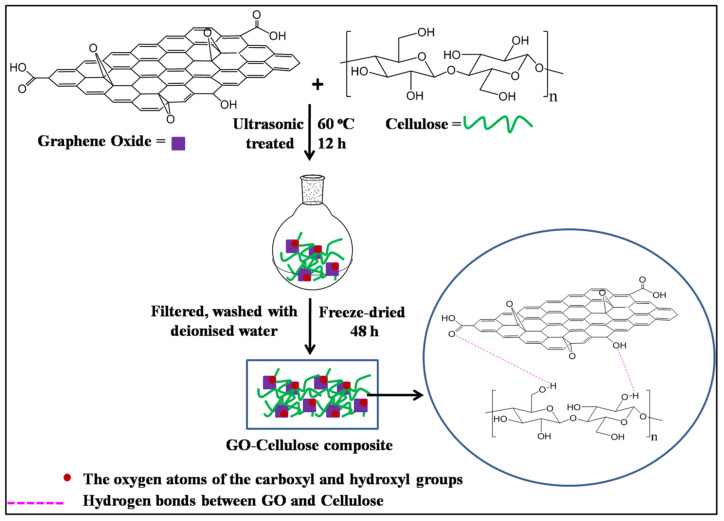
Preparation route of GO–cellulose composite.

The GO–cellulose composite was prepared by the ultrasonication method as per the following procedure (Scheme 1) [33,34]:1.0 g extracted cellulose was added into homogeneous GO (100 mL, 10 mgmL^−1^) aqueous dispersion and then mixed uniformly under vigorous ultrasonication (40 kHz, 80 W) at 60 °C for 12 h. Afterfiltration and washing with deionizedwater, the obtained solid sample wasfreeze-dried using a lyophilizer (Spencers) for 48 hundera vacuum.

### 2.5. Characterization

The IR spectra were recorded withpellets of KBronFT-IR spectrometer (PerkinElmer) in the range of 400–4000 cm^−1^ with 16 scans. Powder XRD patterns were obtained by CuKα radiation (λ = 0.154056 nm) with a step of 0.02^◦^/s in the continuous scan mode (XRD-Rigaku, Germany). The morphology of samples was observed with FE-SEM (Zeiss Gemini, Germany). The samples’ thermal gravimetric analysis (TGA/DTG) was calculated in a N_2_ atmosphere by EXSTAR TG/DTA 6300 instrument. The temperature range was running from 35 to 700 °C with a heating rate of 10 °C/min. The specific surface area and the pore volume were calculated by BETanalysisvia the nitrogen adsorption–desorption method at 77 K (Quantachrome Instruments). The analysis of phenol concentrations was evaluated with UV–Vis spectrophotometer (Shimadzu UV-2600).

### 2.6. Phenol Adsorption Experiments

Thephenol adsorption from aqueous media was examinedand optimized using different parameters which altered the adsorption phenomenon. Adsorption studies were performedin batch mode at pH 7 and room temperature, i.e., 30 ± 2 °C (unless otherwise mentioned). The pH effect was investigated in pH range 3.0–11.0, and the pH was adjusted with 0.1N HCl or 0.1N NaOH. The pH was measured using a pH meter (Ecoscan, EUTECH Instruments, Singapore). The effects of contact time, amount of GO–cellulose, and temperature on the adsorption performance of phenol onto GO–cellulose composite were also studied.For this purpose, 30 mL of phenol with 30 mg/L concentration was taken in a magnetically stirred (250 rpm) round-bottom flask, and the adsorption amount of phenol was determined. After desired adsorption periods, adsorbents were separated from solutions, and phenol concentrations were evaluated using UV-Vis spectroscopy at 270 nm (Shimadzu UV-2600). The adsorption amount of phenol *q_e_* (mg/g), expressed as the amount of adsorbate adsorbed per unit mass of the adsorbent in specified time t, was determined by using the following equation:

$$q_{e}=\left(C_{0}-C_{e}\right)V/m$$

*C_o_* and *C_e_* (mgL^−1^) refer toinitial and equilibrium phenol contents, respectively. *V* (L) is the volume of the adsorption solution, and m (g) is the mass of the adsorbent used.

## 3. Results and Discussion

### 3.1. Characterization of Extracted Cellulose, GO, and GO–Cellulose Composite

**Figure 1:**
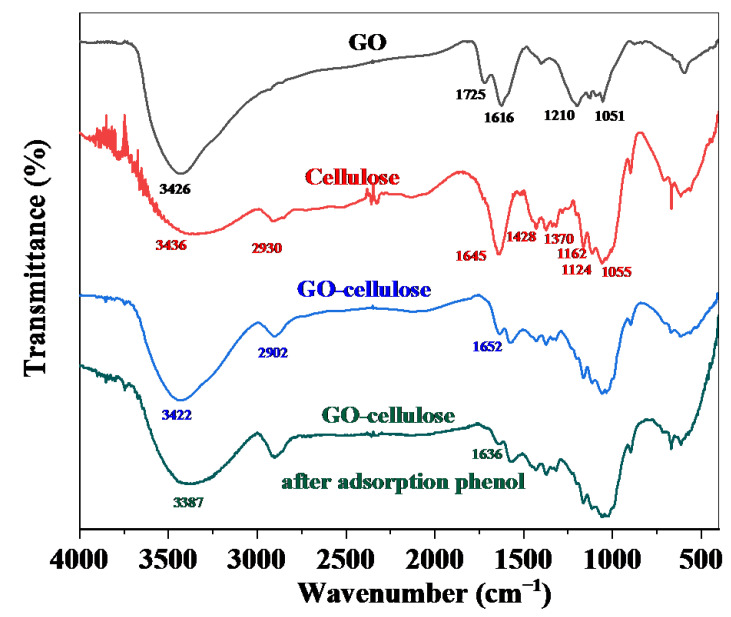
FTIR spectra of GO, extracted cellulose, and GO–cellulose composite.

The FTIR spectra of GO, extracted cellulose, GO–cellulose, and GO–cellulose after adsorption phenol aredelineated in Figure 1. The GO powder exhibits peaks at 3426, 1725, 1616, 1210, and 1051 cm^−1^ corresponding to –OH stretching motions, C=O stretching vibrations of the -COOH groups, C=C of aromatic skeletal stretching vibrations, C–OH stretching, and C-O-C stretching vibrations, respectively. These absorption peaks reveal the presence of oxygen-rich hydroxyl, ether, and carboxyl functionalities in the GO [35]. The extracted cellulose presented the characteristic IR band at 3300–3500 cm^−1^ due to –OH stretching vibrations [18,36] and the band around 2930–2840 cm^−1^ due to the C–H stretches [18], while the peaks at 1645, 1428, and 1370 cm^−1^ correspondedto –OH bending, C-OH stretching, and C-H bending vibrations [18,26,36], respectively. Moreover, the peaks at 1162, 1124, and 1055 cm^−1^ revealed the cellulosic structure’s C-O-C and C-O linkage-based backbone [18,26]. Thus, thesecharacteristic peaks suggest that the cellulose was successfully extracted from ground nut shells. In the case of the GO–cellulosecomposite, the characteristic absorption bands of GO and extracted cellulose appeared in the FTIR of GO–cellulose. The band’s intensityof 2902 cm^−1^ slightly increased after GO mixing with extracted cellulose, corresponding to the cellulose’s C–H stretching vibration [36]. Moreover, in the GO–cellulose composite the band at 3422 and 1652 cm^−1^ corresponds to the shift of the 3436 and 1725 cm^−1^ band ofcelluloseand GO, which confirms the hydrogen bond formation between -OH groups of extracted cellulose and -COOH groups of GO [18]. Thus, the FTIR results indicate that GO successfully interacted with the extracted cellulose. The FTIR spectra of spent GO–cellulose was also recorded, and it was found that after adsorbing phenol, the bands at 3387 cm^−1^ (–OH stretching vibrations) and 1636 cm^−1^ (C=O stretching vibrations of the -COOH groups) correspond to the shift of the 3422 and 1652 cm^−1^ band of the GO–cellulose composite, which reveals the hydrogen bonding interactions between –OH and -COOH groups of the composite and phenol [37].

### 3.2. X-ray Diffraction (XRD) Analysis

**Figure 2:**
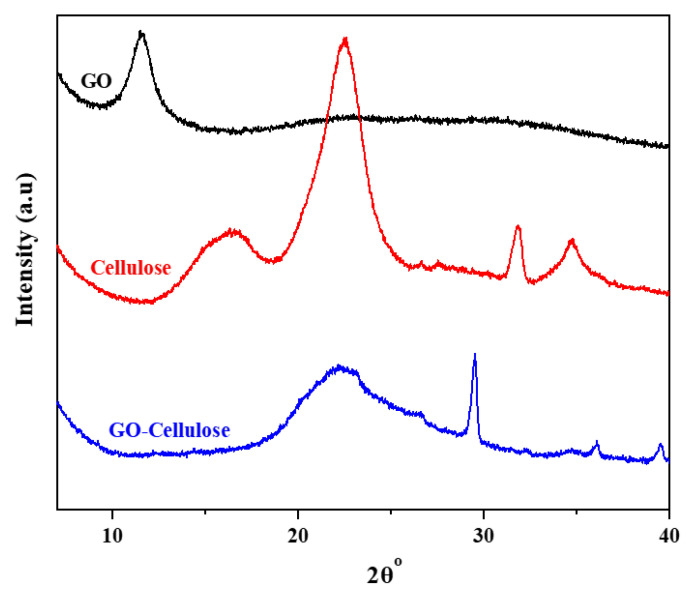
XRD patterns of GO, extracted cellulose, and GO–cellulose composite.

The XRD patterns of GO, extracted cellulose, and GO cellulose are shown in Figure 2. GO has a characteristic peak at 2θ = 11.4° corresponding to the (002) plane which is attributed to the existence of covalently bound oxygen-containing functional groups and the quantity of absorbed water [13,38]. Extracted cellulose presents characteristic peaks at 2θ = 16.3°, 22.5°, and 34.8°, corresponding to 110, 200, and 004 crystal planes, respectively [39]. The peak at 31.8° is due to moisture content, which is supported by the literature-reported water peak in XRD [40,41].The diffraction peaks of the GO–cellulose composite observed at 2θ = 22.2°, 29.5°, and 35.9° are almost consistent with those of the pure extracted cellulose. The characteristic peak of GO at 11.4° disappears completely in the GO–cellulose composite, indicating the removal of oxygen-containing groups from GO, which formed the hydrogen bonding with the -OH groups of cellulose, illustrating the successful combination of GO and cellulose [13]. Moreover, the hydrogen bond was also confirmed between the GO and extracted cellulose.Furthermore, the peak areas at 2θ = 16.3° and 22.2° confirm the amorphous nature of the cellulose and GO–cellulose composite. In addition, compared with the crystalline regions, the amorphous regions of GO–cellulose composites offer higher chemical reactivity [26].

### 3.3. FE-SEM andEDSAnalyses

**Figure 3:**
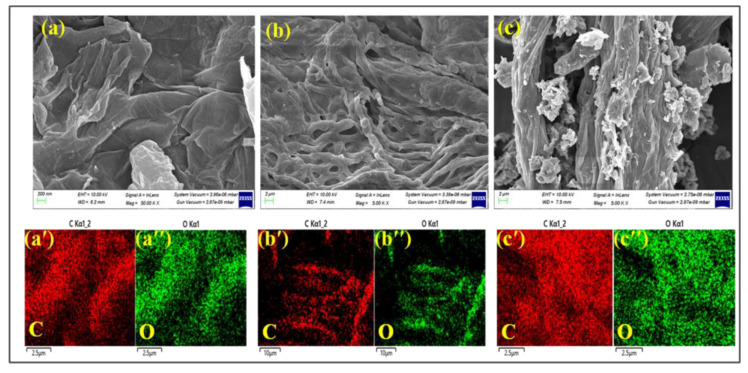
FE-SEM image (**a**) and elemental mapping of C (**a’**) and O (**a”**) of GO, (**b**) elemental mapping of C (**b’**) and O (**b”**) of extracted cellulose, and (**c**) elemental mapping of C (**c’**) and O (**c”**) of GO–cellulose composite.

The morphologies of theGO, extractedcellulose, and obtained GO–cellulose compositewere studied by FE-SEM, and the elemental mapping is shown in Figure 3a–c. GOexhibits its standard layered structures, as shown in Figure 3a. GO has featured a layer-by-layer network with dense stacking of GO sheets [19]. Cellulose presentedafibrillar structure, as shown in Figure 3b [42]. Notably, the composite material’s surface morphology reveals the GO sheets’ appearance on the extracted cellulose surface, as shown in Figure 3c. Thus, the fibrillar structure of cellulose wrapped by the GO sheets indicates the interaction between GO and extracted cellulose.

**Figure 4:**
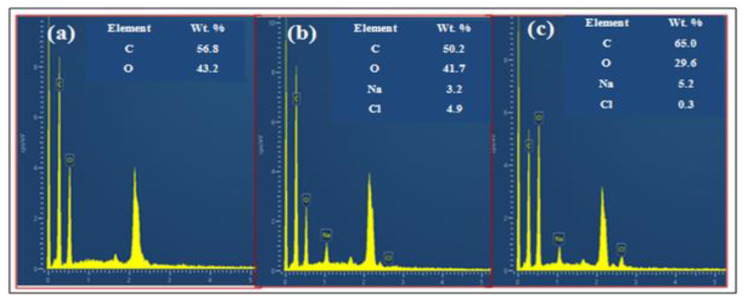
EDS spectra of GO (**a**),extracted cellulose (**b**), and GO–cellulose composite (**c**).

Further, the dispersion of GO into the extracted cellulose was analyzed by energy dispersion X-ray spectroscopy (EDS) to identify the present elemental composition analytically. Figure 4 delineates the EDS spectrum of the GO, extracted cellulose, and prepared GO−cellulose composite. The major elements present in GO, extracted cellulose, and GO−cellulose are illustrated in Table 1. The results of the EDS investigation specify that the atomic percentage of carbon increased in the GO−cellulose composite after mixing the GO with extracted cellulosecompared to GO and extracted cellulose. The improvement % of carbon also suggests the GO−cellulose composite formation.

### 3.4. Thermogravimetric Analysis (TGA) and Differential Thermal Gravimetry (DTG)

**Figure 5:**
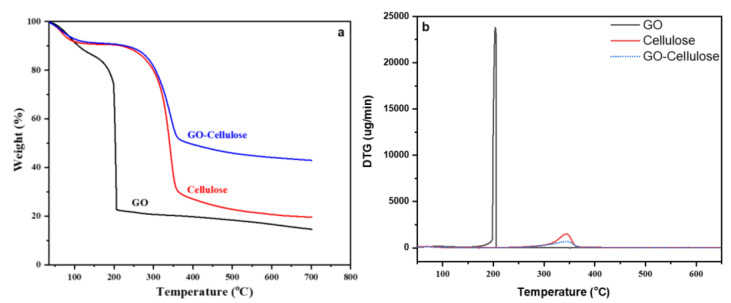
TGA (**a**) and DTG curve of GO (**b**), extracted cellulose, and GO–cellulose composite.

The TGA and DTG curves of GO, extracted cellulose, and the GO–cellulose compositeare shown in Figure 5. The temperature relating to the peak height of the DTG curve is defined as T_max_ (the temperature at which the weight loss rate is maximum). The T_max_s of GO, extracted cellulose, and GO–cellulose were203, 341, and 346 °C, respectively, as shown in Figure 5b. As presented in the TGA curves (Figure 5a), for all samples a slight weight loss was observed before 100 °C due to the removal of physically absorbed water. GO showed drastic weight loss at around 200 °Cdueto the pyrolysis of oxygen-containing functional groups. Pure extracted cellulose exhibited major degradation with weight loss (60.70%) in the temperature range of 240 °Cto 522 °C. This is due to the breakdown of cellulose by pyrolysis reactions [43]. Interestingly, the GO–cellulose did not show weight loss around 200 °C, indicating that the GO layers’ functional groups interacted well with the cellulose functional groups. However, the GO–cellulose composite showed a major weight loss in the temperature range of 266–465 °C, with a maximum decomposition peak at approximately 346 °C. Moreover, the GO–cellulose composite shows a much higher residue than pure cellulose due to the substantial interactions between GO and extracted cellulose through carbonization [18,44]. Based on TGA and DTG results, the thermal stability of the GO–cellulose composite slightly improved compared to extracted cellulose.

### 3.5. Pore Specifications and Surface Area Analysis

The GO–cellulose composite’s porous nature and specific surface area were further examined by BET analysis, and the important parameters are summarized in Table 2. The BET specific surface area of GO–cellulose (up to 6.042 m^2^/g) is higher than that of the cellulose (1.023 m^2^/g). Moreover, the total pore volume, micropore volume, and pore size of GO–cellulose were larger than those of cellulose. This is consistent with the loose porous structure of GO–cellulose. As expected, GO–cellulose has a large BET surface area and pore volume. These results indicate that the high surface area of the GO–cellulose composite may be responsible for phenol adsorption [45].

### 3.6. Adsorption of Phenol Onto GO–Cellulose Composite

#### 3.6.1. Effect of Contact Time

**Figure 6:**
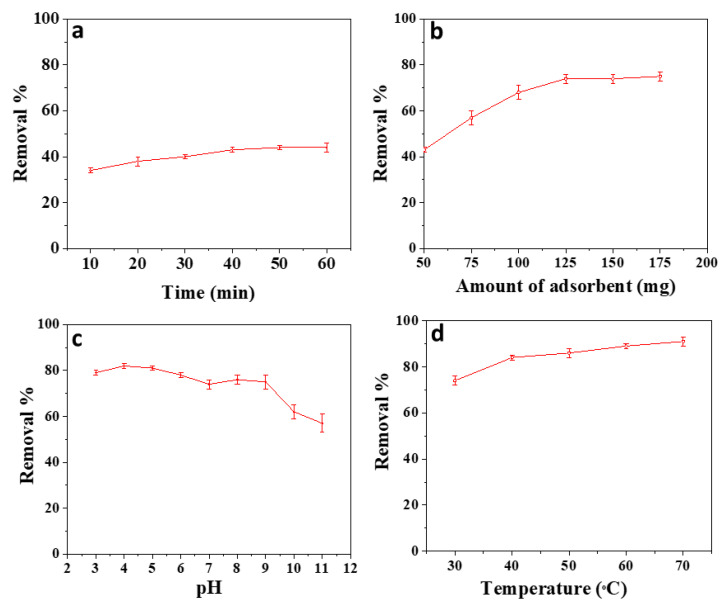
(**a**) Effect of contact time on adsorbent (GO–cellulose composite) of phenol = 30 mg L^−1^; pH 7; adsorbent = 50 mg; and temperature = 30 ± 2 °C. (**b**) Effect of adsorbent dose on adsorption of phenol = 30 mg L^−1^; pH 7; shaking time = 40 min; and temperature = 30 ± 2 °C. (**c**) Effect of pH on the adsorption of phenol onto GO–cellulose composite = 30 mg L^−1^; adsorbent = 125 mg; shaking time = 40 min; and temperature = 30 ± 2 °C. (**d**) Effect of temperature on the adsorption of phenol onto GO–cellulose composite = 30 mg L^−1^; adsorbent = 125 mg; shaking time = 40 min; and pH 7.

Figure 6a shows the effect of contact time on phenol adsorption percentage by the GO–cellulose composite. To study the effect of contact time on the adsorption of phenol, a 30 mL solution of phenol with 30 mg/L concentration containing 0.050 g GO–cellulose was shaken mechanically for varying times (10–60 min) at room temperature and pH 7. The percentage of phenol removed by GO–cellulose composites increased quickly during the initial 20 min and then slowly increased further, reaching equilibrium within 40 min. The initial fast removal rate is likely due to the accessibility of unoccupied locations on the surface of the GO–cellulose composite. With further increasing time, the availability of the remaining unoccupied sites decreased, so phenol adsorption tended to slow [8,37]. As a result, the 43 ± 1% phenol removal was observed in 40 min with 7.74 mg/g adsorption capacity of the GO–cellulose composite. Based on the effect of time results, 40 min was designated as the optimal equilibrium time for additional tests.

#### 3.6.2. Effect of GO–Cellulose Composite Dose

The effect of the GO–cellulose compositeamount on the removal percentage of phenol after 40 min of incubation is shown in Figure 6b. The GO–cellulose composite′s different amounts in the range of 0.050–0.175 g (0.050 g, 0.075 g, 0.100 g, 0.125 g, 0.150 g, and 0.175 g) were added to the 30 mL solution containing 30 mg L^−1^ of phenol to obtain the optimum dose of the adsorbent. As presented in Figure 6b, phenol’s removal percentage increased with the GO–cellulose amount due to the more available surface area for adsorption. It was observed that a 74 ± 2 % phenol removal was achieved when the GO–cellulose amount was 0.125 g/30 mL (adsorption capacity = 5.328 mg/g). It is clear from Figure 6b that 0.125 g showeda nearly equal adsorption performance as 0.150 g and 0.175 g. Hence, 0.125 g of adsorbent was chosen as the optimum amount for further studies.

#### 3.6.3. Effect of pH

Further, the effect of pH was analyzed on the adsorption of phenol on the GO–cellulose composite. The pH is an essential parameter for the adsorption of phenol from aqueous solutions, as it affects the surface charge of the adsorbent, the solubility, and the degree of ionization of phenol. The impact of initial pH on phenol adsorption was evaluated at 30 °C at variable pH ranges of 3–11 for the initial concentration of phenol 30 mg/L containing 0.125 g GO–cellulose. Figure 6 exhibits that the phenol removal was maximal and remained almost unaltered when the original pH of the phenol solution ranged from 3 to 9. Further, a sudden decrease in phenol adsorption was observed withincreasing pH. Note that phenol is weakly acidicwith pKa ≈ 9.89, therefore below this pH, phenol acts as a neutral molecule, and above this value is dissociated and formed as phenolate anions. The adsorption decreases after pH 9 due to the ionization of phenol molecules [8].

#### 3.6.4. Effect of Temperature

The temperature effect on phenol adsorption ofthe GO–cellulose composite was examined at five temperatures, i.e., 30, 40, 50, 60, and 70 °C. To study the effect of temperature on the adsorption of phenol, a 30 mL solution of phenol at an initial concentration of 30 mg/L andpH 7 containing 0.125 g GO–cellulose was shaken mechanically at varying temperatures (30–70 °C).The results are given in Figure 6d, and the % removal of phenol and adsorption capacity (q_e_) rose from 74% and 5.328 mg g^−1^ to 91% and 6.552 mg g^−1^ when the temperature increased from 30 to 70 °C. These results demonstrate that phenol’s adsorption is an endothermic process [46]. The removal% of phenol and the details of the reaction conditions are illustrated in Table 3. It is clear from Table 3 and Figure 6 that the best optimumreaction conditions for the adsorption of phenol by the synthesized composite are those mentioned in entry 21 of Table 3, resulting in 86 ± 2% phenol removal.

#### 3.6.5. The Effect of the Initial Concentration of Phenol

**Figure 7:**
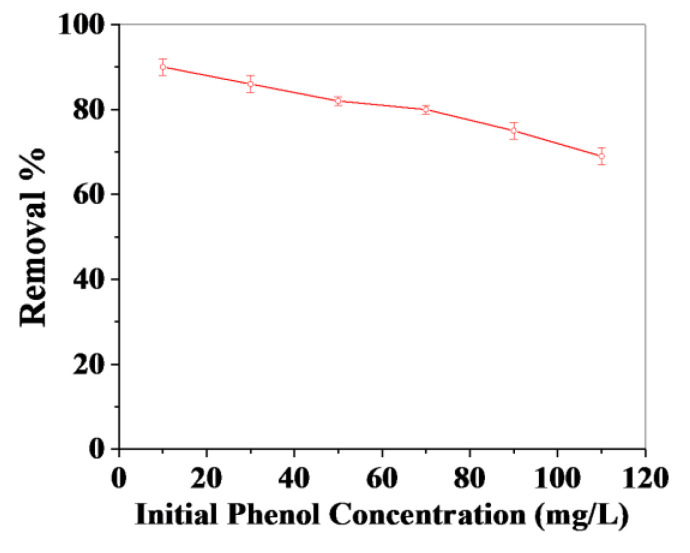
Effect of the initial concentration of phenol on the adsorption performanceof phenol on GO–cellulose composite:adsorbent = 125 mg; shaking time = 40 min; pH = 7; and temperature = 50 °C.

Figure 7shows the effect of the initial concentration of phenol on the adsorption of phenol on the GO–cellulose composite. In our investigation, the removal percentage of phenol was studied by varying the initial phenol concentration from 10–110 mg/L (10 mg/L, 30 mg/L, 50 mg/L, 70 mg/L, 90 mg/L, and 110 mg/L) at optimized reaction conditions, i.e., at constant time (40 min), constant pH (7), constant adsorbent quantity (0.125 g/30 mL), and at constant temperature (50 °C).As presented in Figure 7, phenol’s removal percentage decreased from 90% to 69% with increasing initial concentration of phenol from 10–110 mg/L. A similar observation was reported by Das et al. [47,48] for the adsorption of phenol using rice husk ash and guava tree bark as adsorbents.

## 4. Comparison of Phenol Removal with Various Adsorbents Reported in the Literature

The adsorption capacity of GO–cellulose for removing phenol from aqueous solutions was compared with other adsorbents studied in the literature. Experimental data from the current investigation are comparable to reported values. The GO–cellulose composites in this study demonstrate large adsorption capacities compared to many otheradsorbents. Although some adsorbents have already shown greater adsorption capacities for phenol removal, Table 4 summarizes the adsorption capacities of various types of adsorbents for phenol under more or less similar experimental conditions.

## 5. Adsorption Isotherms and Kinetic Models

The Langmuir and Freundlich isotherms were applied for the adsorption of phenol onto the GO–cellulose composite. The Langmuir and Freundlich isotherms in their linear forms can be given as Equations (1) and (2), respectively [58].

**Figure 8:**
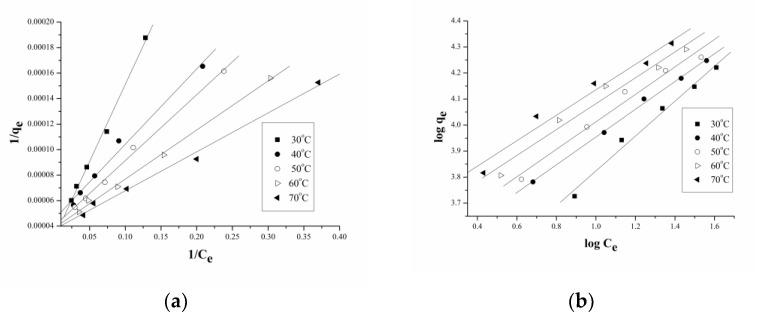
Plots for (**a**) Langmuir and (**b**) Freundlich isotherms at different temperatures.

1/qe = (1/Q°b)(1/Ce) + (1/Q°)
(1)

logqe = log Kf + 1/n log Ce
(2)

where Q°, b are Langmuir parameters and 1/n, Kf are Freundlich parameters. The data were fitted to both the isotherm models and the plots obtained were straight lines (Figure 8a,b). The values obtained for the correlation coefficient (R2) clearly indicate that the data were better fitted to Langmuir isotherms at all temperatures (Table 5). A similar observation was reported by Singh et al. [59] for the adsorption of Rhodamine B dye using powdered banana peel as the adsorbent. The better applicability of the Langmuir model was also reported for the adsorption of brilliant green dye on activated carbon prepared using banana peels [60].

**Figure 9:**
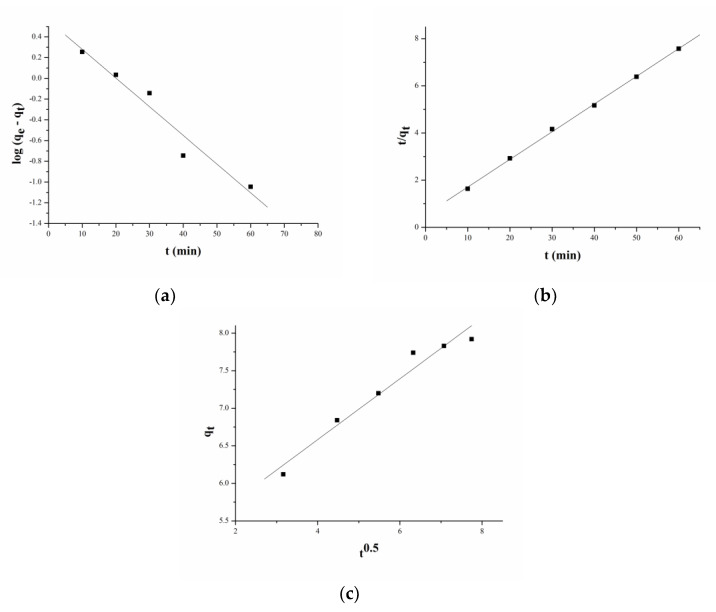
Plots for (**a**) pseudo-first-order, (**b**) pseudo-second-order, and (**c**) intra-particle diffusion models for adsorption of phenol on GO–cellulose composite.

The pseudo-first-order, pseudo-second-order, and intra-particle diffusion models were applied to analyze the kinetics of adsorption of phenol onto the GO–cellulose composite [61]. The plots obtained for the different kinetic models were straight lines (Figure 9a–c). The better applicability of the pseudo-second-order model was suggested by the values of correlation coefficients (Table 6). The enhanced applicability of the pseudo-second-order model was also reported by Kumar and Gupta [62] and Yun et al. [63] for the adsorption of naphthalene on activated carbon from sawdust and porous carbon materials, respectively. The plot for the intra-particle model suggests the occurrence of different adsorption mechanisms at the same time [62].

## 6. Conclusions

The GO–cellulose composite was prepared successfully using ultrasonication, and the adsorption properties for phenol were investigated. The results of FTIR, XRD, and EDX presented the successful interaction between the functional groups of GO and extracted cellulose. The TGA and DTG analyses show a slight improvement in the thermal stability of the GO–cellulose composite compared to extracted cellulose. The GO–cellulose composite exhibited good adsorption capacity toward phenol, and the adsorption equilibrium can be achieved within 40 min. Based on the phenol adsorption results, the optimal conditions were investigated.The adsorption process showed that in optimum conditions, 86 ± 2% of phenol was removed in 40 min with an adsorption capacity of 6.192 mg g^−1^. Moreover, the adsorption experimental data fit well to the pseudo-second-order kinetic model and Langmuir isotherm model. Therefore, these results indicate that the prepared GO–cellulose composite can be a potentially effective absorbent for removing phenol from an aqueous solution.

## Figures and Tables

**Table 1:** The element compositions in GO, extracted cellulose, and GO−cellulose composite.

Sample	Carbon	Oxygen	Sodium	Chlorine
GO	56.8	43.2	-	-
Cellulose	50.2	41.7	3.2	4.9
GO−cellulose	65.0	29.6	5.2	0.3

**Table 2:** Specific surface area and porous structure parameters of extracted cellulose and GO–cellulose composite.

Sample	*S_BET_* (m^2^/g)	V_T_ (cm^3^/ g)	V_mic_ (cm^3^/ g)	D_p_ (nm)
Cellulose	1.023	1.28	0.001	3.146
GO–cellulose	6.042	4.094	0.003	4.757

*S_BET_*: BET surface area; V_T_: total pore volume; V_mic_: microporevolume; D_p_: pore diameter.

**Table 3:** Details of reaction conditions and the % removal of phenol under each particular condition.

Entry No.	Time (Min)	Dose (g)	pH	Temperature (°C)	Phenol Removal (%)
1	10	0.050	7.0	30	34 ± 1
2	20	0.050	7.0	30	38 ± 2
3	30	0.050	7.0	30	40 ± 1
4	40	0.050	7.0	30	43 ± 1
5	50	0.050	7.0	30	44 ± 1
6	60	0.050	7.0	30	44 ± 2
7	40	0.075	7.0	30	57 ± 3
8	40	0.100	7.0	30	68 ± 3
9	40	0.125	7.0	30	74 ± 2
10	40	0.150	7.0	30	74 ± 2
11	40	0.175	7.0	30	75 ± 2
12	40	0.125	3.0	30	79 ± 1
13	40	0.125	4.0	30	82 ± 1
14	40	0.125	5.0	30	81 ± 1
15	40	0.125	6.0	30	78 ± 1
16	40	0.125	8.0	30	76 ± 2
17	40	0.125	9.0	30	75 ± 3
18	40	0.125	10.0	30	62 ± 3
19	40	0.125	11.0	30	57 ± 4
20	40	0.125	7.0	40	84 ± 1
21	40	0.125	7.0	50	86 ± 2
22	40	0.125	7.0	60	89 ± 1
23	40	0.125	7.0	70	91 ± 2

**Table 4:** A comparative study of the adsorption behavior of GO–cellulose composites and other adsorbents on phenol.

Adsorbents	q_e_(mg/g)	Ref.
PSCC (palm seed coat activated carbon)	18.3	[10]
Fe_3_O_4_/PRd composite	4.98	[21]
CA/APS-derived AC composite	45.0	[49]
ACK1 activated carbon	17.83	[50]
ACK2 activated carbon	45.49	[50]
Activated bentonites	4–16	[51]
Banyanroot activated carbon (BRAC)	26.95	[52]
Corn husk activated carbon	7.8	[53]
Modified fly ash	4.79	[54]
BioZheolith	2.354	[55]
Surfactant-modified alumina	6.64	[8]
NH2-MIL-101 (Al)	0.76	[8]
Bagasse fly ash	0.060	[8]
Rice husk	0.0022	[8]
Petroleum coke	6.01	[8]
Pyrolyzed sewage sludge	5.56	[8]
Activated coal	1.48	[56]
Ultrasonic assisted graphene oxide nanosheet	10.23	[57]
GO–cellulose	6.192	This study

**Table 5:** Langmuir and Freundlich parameters for the adsorption of phenol.

	LANGMUIR PARAMETERS						FRENDLICH PARAMETERS				
Term	Values					Term		Values			
	30 °C	40 °C	50 °C	60 °C	70 °C		30 °C	40 °C	50 °C	60 °C	70 °C
B	0.025	0.0833	0.0800	0.100	0.0133	1/n	0.6696	0.5331	0.5277	0.4969	0.4891
R ^2^	0.9957	0.9873	0.9963	0.9974	0.9923	R ^2^	0.9863	0.9867	0.9773	0.9679	0.9662
Q°	3.33 × 10^4^	2.00 × 10^4^	2.50 × 10^4^	2.52 × 10^4^	2.53 × 10^4^	K_f_	1426.59	2635.72	3052.81	3847.68	4430.98

**Table 6:** Parameters for kinetic and intra-particle diffusion models.

Pseudo-First-Order		Pseudo-Second-Order		Intra-Particle Diffusion	
Rate Constant	R^2^	Rate Constant	R^2^	Rate Constant	R^2^
6.37 × 10^−2^	0.9499	2.58 × 10^−2^	0.999	4.05 × 10^−1^	0.9797

## Data Availability

The data presented in this study are available on request from the corresponding author.
